# Impact of policies regulating foreign physician migration to Switzerland: a modelling case study in anaesthesia

**DOI:** 10.1186/s12913-015-0867-3

**Published:** 2015-05-22

**Authors:** Guy Haller, Christophe Combescure, Chantal Mamie, Davide Zoccatelli, François Clergue

**Affiliations:** Division of Anaesthesia, Department of Anaesthesiology, Pharmacology and Intensive Care University Hospitals of Geneva, 4, rue Perret-Gentil, 1211 Genève 14, Switzerland; Health Services Management and Research Unit, Department of Epidemiology & Preventive Medicine, Monash University, Melbourne, Australia; CRC and Division of Clinical Epidemiology, Department of Health and Community Medicine, University of Geneva, University Hospitals of Geneva, Geneva, Switzerland

## Abstract

**Background:**

Several countries have developed policies that restrict or limit duration of stay, clinical privileges or the number of residency permits allocated to migrating physicians. Switzerland is currently preparing a new law limiting overall foreign immigration. The impact of such restrictive policies is currently unknown. In a case study of anaesthesia care in Switzerland we modelled, trends in the size of physicians’ workforce until 2024, following the implementation of a strict quota policy for foreign medical trainees.

**Methods:**

We developed a computer-based Markov model with Monte-Carlo simulations to project, in the context of a strict quota policy for foreign trainees, supply and demand for anaesthesia positions until 2024. We used data from a cross-sectional study performed in the French- and Italian-speaking cantons of Switzerland and the Health dataset from the OECD.

**Results:**

With 8 to 12 (95 % CI 4–20) anaesthetists retiring per year, the implementation of strict quotas of foreign graduates would result in a 38 % decrease in the number of anaesthetists in intermediary (senior registrars) positions by 2024. This decrease would be particularly important in district hospitals where nearly half (49 %) of the non-Swiss anaesthetists are practising. Swiss graduates are unlikely to balance the shortage. Despite efforts by Swiss universities to increase the number of medical graduates, their number has dropped from 10.5 to 9.7/100 000 inhabitants between 2000 and 2012, due to the growth of the population.

**Conclusions:**

This case study in Latin Switzerland shows that a restrictive policy limiting foreign immigration of trainees would result in a major deficit in the number of anaesthetists available to meet population needs. These aspects should be carefully considered when countries develop restrictions and limitations of foreign immigration.

## Background

In many countries, growing efforts are devoted by healthcare authorities to the provision of high quality of care to the population. This implies that an appropriate balance between healthcare needs and healthcare workforce provision is maintained. Several studies have for instance demonstrated that there was a significant correlation between physician and nurse density and improved patient outcomes [1–3]. This is why forecasting models have been developed to ensure that this balance is maintained [4]. There are two types of forecasting models: demand-based models that inform need by examining the amount of health care services needed in the future, based on projected size, demographic and healthcare profile of the population and supply projection models that estimate workforce needs to maintain current standards and volume of services per capita [5]. While both forecasting models are used across countries, the supply-side model is the most commonly used method, particularly for physicians [6]. Usually, a defined ratio of physicians per 1000 inhabitants is determined and adjusted to the forecasted population increase (or decrease) accordingly. Then, the number of students allowed to enter medical schools is fixed according to the forecasted need. This method named the “numerus clausus”, is in force in nearly all countries part of the Organisation for Economic Co-operation and Development (OECD), except Ireland and Austria [7].

Despite its wide dissemination, this approach has several weaknesses. Population forecasting models provide only estimates with confidence intervals and not exact numbers. Thus, the exact number of physicians to be trained in medical schools cannot be defined accurately. Furthermore, medical careers’ and speciality choices are determined by a mix of random conditions such as gender, social status, income, perceived burden and working times [8–11]. As a result, even if the appropriate number of physicians to be trained to respond to population needs could be accurately forecasted, major imbalances would still persist, some medical specialities being more popular than others. This is why an increasing number of countries rely on foreign trained physicians’ immigration to compensate for temporary or persistent physician shortage. This has recently been referred to as a “quick and inexpensive fix” for under-planning of workforces [7]. The report on Healthcare workforce in OECD countries (period 1995–2005), shows that for instance Australia, Canada, New Zealand, Norway, the United Kingdom and Switzerland are the six OECD countries where physician immigration rates exceed medical graduation rates. With 9.7 new medical graduates per 100,000 inhabitants in 2012, Switzerland is below the median of 10.4 graduates per 100 000 residents of other OECD countries. Unsurprisingly, Switzerland has become increasingly dependent on foreign medical trainees, particularly in hospitals and surgical/anaesthetic specialties [12]. Currently 32.6 % of anaesthetists and surgeons trainees had their medical diploma delivered outside Switzerland [13].

Despite high needs of foreign medical graduates, a number of countries have implemented restrictive policies that limit either duration of stay, clinical privileges or the number of residency permits allocated to migrating physicians. For instance, the United States have developed a ‘cultural exchange visa’ that allows foreign graduates to stay for only a limited period of time before being required to return home for a two-year period after which he/she is entitled to apply for re-entry again [14]. Switzerland is considering the implementation of a restrictive quota policy for new immigrants, including skilled workers. A strictly predefined number of working and residency permits would be delivered to foreigners, whenever planned residency exceeds four months [15]. Such limitations to skilled workers mobility may have detrimental effects on the overall size of the physician healthcare workforce, particularly in countries that highly rely on foreign immigration. The true impact of such policies is however currently unknown, particularly in specialties like anaesthesia known to be sensitive to workforce containment policies [8, 16].

The purpose of this study was therefore to model the impact of a restrictive policy based on strict annual quotas of residency permits for migrating physicians in a case study of anaesthesia in Switzerland.

## Methods

### Study design

Data were extracted from a cross-sectional study assessing all anaesthetists (in training or certified) practicing anaesthesia as a main activity, in public or private hospitals in all the French and Italian speaking cantons of Switzerland [17]. In order to assess all trainees including those from foreign countries, participants were included through local professional organisations and hospital lists prepared in each canton by two coordinators, one for private and the other for public hospitals. While the original questionnaire sent to participants included 103 items gathering information on demographic characteristics, professional activity and satisfaction, for the modelling study we used a restricted number of variables. These were age, gender, type of professional training, country of origin and diploma, professional activity, type of institution, position, activity ratio, professional life plan and desired retirement age. Details of the questionnaire used for the study are provided in a previous publication [18]. To assess density and other variables requiring population data, we used information available from the OECD healthcare database and from the Swiss Federal Institute of Statistics in Neuchâtel.

University and hospital human research ethics committee approval was not required for this study, as no patient health-related or sensitive information were collected.

### Statistical analysis

In our study context, inflow was based on fixed constraints (quotas of foreign physicians allowed to practice in Switzerland). Therefore we could not use traditional inflow or combined inflow/outflow models [4, 19]. We developed a computer-based Markov model to project the number of anaesthetists and their respective position (in training, transition, fixed or retired) until 2024. In Markov models, transition probabilities from one state to another are estimated according to a number of factors. They are particularly appropriate in workforce studies forecasting human labour market according to a number of predefined constraints [20, 21]. In our model, we considered that factors affecting demand were: official and desired retirement age (when expressed), gender, demographic distribution of existing workforce, number of long term positions available (consultant in a private or public hospital, leading positions in public hospitals, office-based anaesthesia) and age of diploma. The supply-side assumption of the model was that, according to a strict return policy, fixed quotas of foreign trainees entering anaesthesia workforce would be implemented and remain constant over the years. Transition probability between training position (internship), transition positions (senior registrars) and fixed positions (consultants in a private or public hospital, leading positions in public hospitals, out-office positions) were determined by the number of individuals in training, the duration of training, favoured retirement age, gender and number of part/full time positions. Because these factors are interlinked, we used Monte Carlo simulations. These are based on mathematical algorithms which allow the construction of hypothetical cohorts that integrate the coupling of interlinked factors. These factors define the transition probabilities to move from one state to another in the Markov model [22]. Therefore Monte Carlo simulations are particularly sound for coupled variables with several degrees of freedom. In our study transition probabilities from one state to another (i.e. fixed position to retirement) was conditioned by several coupled factors such as age, gender, part/full time work, individual preferences.

Our model’s assumptions were that quota policies would limit training positions to a constant proportion of foreign trainees and that senior positions (fixed positions) would constantly be filled by the existing pool of anaesthetists in transition positions (senior registrars). Results are reported as absolute numbers with 95 % CI.

**Fig. 1:**
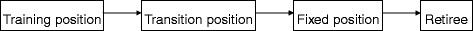
Diagram of the four-state Markov model used to predict the distribution of anaesthetists

Socio-demographic characteristics and professional activity were described using frequency tables and proportions. Continuous variables such as age and working hours were transformed into separate and mutually exclusive categories. For other continuous variables like retirement age we used means and standard deviations. The number and density of medical students graduating from Swiss universities were extracted from the OECD health database (Fig. 1).

Data are reported as absolute numbers and proportions per 100 000 inhabitants [23]. Statistical analyses were performed with Stata/IC 12.0 (Stata-Corp, College station) and S-Plus 8.0 for Windows (Insightful Corp., Seattle).

## Results

### Anaesthetists’ characteristics

Overall, 416 (82 %) of the 506 listed anaesthetists (in training or certified) practising in the Latin (French or Italian-speaking) cantons of Switzerland, provided responses to the survey that could be used for the modelling study. Participants’ characteristics and the distribution of the variables integrated into the model are summarised in Table 1. The proportion of men was 58 % and the majority of anaesthetists was aged between 36 to 55 years. The proportion of trainees was higher amongst anaesthetists from foreign origin (32 %) than amongst Swiss nationals (13 %). While the later practiced mainly in private (25 %) and university-affiliated hospitals (40 %), European and extra-European anaesthetists were mainly working in district hospitals (49 %). There were 24 % of anaesthetists working part-time and desired retirement age was lower in female (60 years) than in male anaesthetists (62 years). For all of them it was lower than the legal retirement age in Switzerland, 64 years for females and 65 years for males. While the official duration of specialty training for anaesthesia in Switzerland is 5 years, the median interval between graduation and certification was in fact 9 years (Table 1).

**Table 1:** Distribution of variables used in the model

Variables	N (%)	Variables	N (%)
Age		Nationality	
≤35 y	97 (24)	Swiss	338 (81)
36-55 y	254 (62)	European	69 (17)
≥56 y	56 (14)	Others	6 (2)
Gender		Type of activity	
Female	171 (42)	All anaesthetists	
Male	239 (58)	Fixed (Consultant, Head; Out-office)	248 (60)
Working hours/week		Transition (senior registrar)	99 (24)
≤50 h	187 (49)	Training (intern, registrar)	66 (16)
51-69 h	161 (42)	Swiss	
≥70 h	41 (9)	Fixed (Consultant, Head; Out-office)	218 (64)
Full/part time		Transition (senior registrar)	80 (23)
Part-time	97 (24)	Training (intern, registrar)	42 (13)
Full-time	315 (76)	European-extra-european	
Desired retirement age		Fixed (Consultant, Head; Out-office)	31 (41)
Males, Years (mean SD)	62 (3.1)	Transition (senior registrar)	20 (27)
Females, Years (mean SD)	60 (2.8)	Training (intern, registrar)	24 (32)
FMH title*		Type of hospital	
No	109 (28)	All anaesthetists	
Yes	288 (72)	University hospital	161 (39)
Duration of specialty training if FMH title*		District hospital	155 (28)
5 years	10 (4)	Private	92 (22)
6 years	32 (12)	Office-based	2 (0)
7 years	42 (16)	Swiss	
8 years	36 (13)	University hospital	133 (40)
9 years	31 (12)	District hospital	119 (35)
10 years	36 (13)	Private	82 (24)
11 years	24 (9)	Office-based	2 (1)
12 years	15 (6)	European-extra-european	
13 years	10 (4)	University hospital	28 (38)
14 years	7 (3)	District hospital	36 (49)
15 years and more	25 (9)	Private	10 (13)
		Office based	0 (0)

*Specialty title of the Swiss Medical Association (FMH).

### Modelling study results

**Fig. 2:**
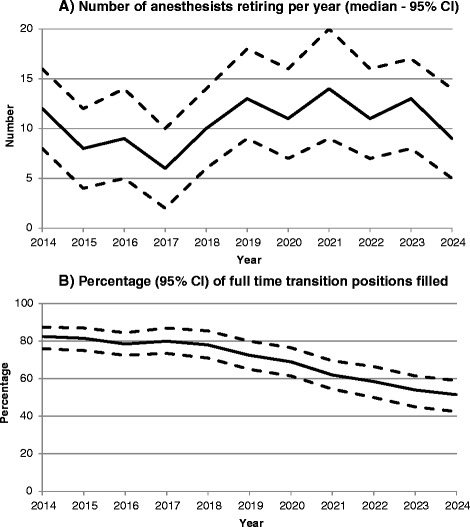
Number of anaesthetists retiring by age category. **a** Number of anesthesists retiring per year (median - 95 % CI). **b** Percentage (95 % CI) of full time transition positions filled

The model shows a progressive increase in the number of anaesthetists retiring by 2024, on average 8 to 12 (95 % CI 4–20) per year (Fig. 2a). If a strict regulation of foreign trainees admitted to work in Switzerland was implemented, a fixed number of physicians would fill the training positions resulting in a progressive imbalance in the number of anaesthetists in transition positions. Data resulting from the modelling process (Table 2) confirm that the number of anaesthetists in transition positions (senior registrars) would be 38 % below the number that would actually be necessary by 2024.

**Table 2:** Distribution of the number of anaesthetists predicted by the model (N, 95 % CI)

Year	Training position	Transition positions	Fixed positions	Retirees
2014	66 (66 to 66)	93 (86 to 98)	244 (241 to 247)	12 (8 to 16)
2015	66 (66 to 66)	92 (85 to 98)	244 (241 to 247)	8 (4 to 12)
2016	66 (66 to 66)	89 (82 to 95)	244 (241 to 248)	9 (5 to 14)
2017	66 (66 to 66)	90 (83 to 98)	244 (241 to 248)	6 (2 to 10)
2018	66 (66 to 66)	88 (80 to 96)	244 (240 to 247)	10 (6 to 14)
2019	66 (66 to 66)	81 (74 to 89)	244 (240 to 248)	13 (9 to 18)
2020	66 (66 to 66)	78 (70 to 85)	244 (240 to 248)	11 (7 to 16)
2021	66 (66 to 66)	70 (61 to 77)	245 (241 to 249)	14 (9 to 20)
2022	66 (66 to 66)	66 (57 to 73)	244 (240 to 249)	11 (7 to 16)
2023	66 (66 to 66)	60 (51 to 68)	244 (240 to 249)	13 (8 to 17)
2024	66 (66 to 66)	58 (49 to 66)	244 (239 to 249)	9 (5 to 14)

**Fig. 3:**
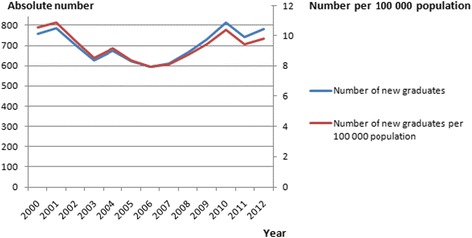
New medical graduates in Switzerland between 2000 and 2012

When modelling the impact of part time work on the total size of the workforce, a gap would already appear by the end of the year 2014 with 10 % of full-time equivalent transition positions not being filled. The gap would reach 50 % (95 % CI 42–59) by the end of 2024 (Fig. 2b). This lack of senior registrars in transition positions would be particularly important in district hospitals where nearly half (49 %) of the non-Swiss anaesthetists are practising (Table 1). This could theoretically be compensated by an increase in the number of medical graduates from Swiss universities. Data extracted from the OECD health database show that this cannot be the case. While the absolute number of new medical graduates increased by 8 % (from 756 to 813) between 2000 and 2010, the growth of the Swiss population resulted in an overall decrease of the rate of new graduates per 100 000 inhabitants, from 10.5 to 10.3 in 2010. This deficit increased even further in 2012, with a rate of 9.7 new graduates per 100 000 inhabitants (Fig. 3).

## Discussion

Using a Markov supply and demand forecasting model, we found that a strict regulation of foreign physicians admitted in anaesthesia training positions in Latin Switzerland would result in a progressive and significant shortage of 38 % in the number of anaesthetists in transition positions by the year 2024. When adding to the model the impact on favoured retirement age of the increasing female to male ratio and the progressive societal shift from full to part-time job, this gap could reach 50 % (95 % CI 42–59) by the end of the projection period. This could have a serious impact on public hospitals but more particularly on district hospitals, where nearly half of the European and extra-European anaesthetists are practising. This case study shows that in countries that heavily rely on foreign physicians to maintain their healthcare workforce balance, the implementation of restrictive policies such as strict quotas (i.e. Switzerland) or return policies (i.e. United States) are likely to cause a significant deficit in physician workforce, particularly in district areas.

While the impact of a restrictive policy on trained physicians’ immigration has not been assessed elsewhere, a number of related publications confirm our findings. In a study on Austrian psychiatrists, Riedel et al. found that the implementation of quotas for different nationalities at entry in medical school was one of the main contributor to the projected 5 % gap between demand and supply in 2030 [24]. In another modelling study on optometric supply in Australia, Kiely et al. forecasted a 21.5 % decline in the proportion of optometrists in active practice if the number of national graduate and immigrants was going to decrease in the future [25].

Therefore, when the provision of locally trained physicians is insufficient to meet population needs, countries should engage in policies facilitating rather than impeding immigration. However, these should be considered only as temporary buffers as such policies are likely to further increase the brain drain already present in numerous countries [26, 27]. Western OECD countries are currently the main destination for medical migrants while developing and East European countries experience a progressive shortage of physicians due to emigration [28]. The brain drain of physicians has a serious negative impact on healthcare systems of countries with a negative immigration balance [29, 30]. Therefore, a number of specific guidelines and codes of good practice have been developed to promote ethical, transparent, fair and mutually beneficial agreements between source and destination countries [14].

Some examples are the Commonwealth Code of Practice for International Recruitment of Health Workers [31] and the ‘Global Code of Practice on the International Recruitment of Health Personnel’ of the World Health Organisation [32]. While such policies are laudable initiatives, their impact on the brain drain of skilled healthcare workers from developing countries remains marginal. Codes of practice are voluntary initiatives of the signatories and compliance is hardly monitored or enforced [33]. Governance in the field of human resources for health remains relatively poor [34].

Interestingly, extensive policies aimed at limiting immigration, while integrating little ethical considerations, are likely to have a more significant impact on brain drain than codes of good practices. In our case study, we forecasted a 50 % (95 % CI 42–59) decrease (adjusted for part-time positions) within 10 years of the number of anaesthetists in intermediary positions (senior registrars), should strict immigration quotas for foreigners be implemented in Switzerland.

It could be argued that immigrants contribute to increase the size of the population and thus, a strict regulation of immigration could maintain the balance of the physician to population ratio. However, this ignores the increased demand of the population for care, particularly in the surgical area. In Switzerland for instance, between 2002 and 2008 there has been a19 % increase in the overall number of core surgical procedures (inpatient and outpatient) [35]. A similar trend has been observed in most OECD countries where healthcare needs have increased [36], particularly for surgical procedures such as hip or knee replacement, with a 10 to 60 % raise between 2000 and 2010. As a result, limiting global immigration is likely to have no impact on the growing need for additional physicians.

To minimise reliance on foreign trained physicians and limit the brain drain of physicians in source territories, countries should engage in policies that increase domestic physician supply. These should not be done only through quota adjustments of medical students [37], but also through initiatives that tend to improve the retention of physicians in the workforce. This could be done by increasing retirement age and working hours. However, these should be in line with both the generation shift and existing laws defining retirement age and regulating the maximum amount of working hours authorized for physicians. In Switzerland, these are set at 50 h a week [38–40].

Another option, in the case of anaesthesia care, could be the implementation of independent anaesthesia nurse practitioners. In Switzerland, nurse anaesthetists are a significant part of the anaesthesia workforce and are allowed to perform a large number of procedures such as drug administration, tracheal intubation, patient monitoring and surveillance. These have to be performed under the strict supervision of a certified anaesthetist. In the near future, they could provide anaesthesia care without any assistance in low risk ASA 1 and 2 patients.

There is to date much controversy as to whether this approach would be detrimental or not to the overall quality of care [41, 42]. Professional organizations are still debating whether or not, they should engage in that direction. Furthermore, the recruitment of nurses can be quite difficult and many countries suffer from a nurses’ workforce shortage. As a result, Switzerland like many other European countries heavily relies on foreign trained nurses to care for its population [43].

Another alternative could be to increase productivity through technological innovation, financial incentives such as fee-for-service payments and the development of day care surgery [44, 45]. Some of these developments are already underway. For instance, an increasing number of surgical procedures in Switzerland are performed on a day care basis, increasing patient turnover in hospitals and overall productivity. Replacing cost-reimbursement by activity-based payments that reward efficiency is another possible method that could enhance productivity [46]. This strategy should be balanced with the increased risk of jeopardizing the overall quality of the service provided, if patient turnover is escalated excessively. In healthcare, productivity cannot be increased perpetually and a minimum staff/patient ratio needs to be achieved in order to minimize the risk of adverse outcomes [1, 3]. This is especially true in anaesthesia, a high risk specialty [47–49].

There are a number of limitations to our study. First, in our model, we assumed that over the years, only fixed and constant quotas of non-Swiss trainees would be allowed to work and live in the country. While this is likely to be the case with the new law, the exact outline of the Swiss quota immigration policy is currently still under consideration. Our model is therefore only one of the several possible scenarios that may result from the new immigration policy. Secondly, the model included only data collected in the French- and Italian-speaking part of Switzerland, limiting the generalisability of study findings. Finally, we lacked information on anaesthetists working in intensive care and could not draw conclusions on this category of healthcare professionals although they contribute to post anaesthesia care. Despite these limitations, the present study is the first one to assess the possible impact of restrictions on skilled immigration in a case study of the specialty of anaesthesia. Future studies should explore the impact of such policies in other medical specialties and other OECD countries that heavily rely on skilled immigration to supply healthcare to their home population. It would also be important to perform such studies in nursing, midwifery and other allied healthcare workforce areas which also often rely on skilled immigration in many countries.

## Conclusions

This case study of anaesthesia care in Switzerland shows that insufficient provision of locally trained medical graduates combined to a policy limiting entry of foreign trainees is likely to cause a major deficit in the number of anaesthetists available to meet population needs. In the context of growing physician shortages in many OECD countries, migration flows can offer temporary solutions to erroneous healthcare workforce planning and increased population needs. These should however only be considered as short-term buffers as they contribute to the brain drain of physicians in countries where they are highly needed. Instead, countries should engage in policies that can create adequate supplies of domestic physicians by enhancing education and training whilst at the same time improving methods of retention of the existing workforce.
